# First molecular determination of herpesvirus from two mysticete species stranded in the Mediterranean Sea

**DOI:** 10.1186/s12917-015-0596-1

**Published:** 2015-11-14

**Authors:** Mar Melero, José Luis Crespo-Picazo, Consuelo Rubio-Guerri, Daniel García-Párraga, José Manuel Sánchez-Vizcaíno

**Affiliations:** VISAVET Center and Animal Health Department, Veterinary School, Complutense University of Madrid, 28040 Madrid, Spain; Veterinary Services, Oceanogràfic, Ciudad de las Artes y las Ciencias, 46013 Valencia, Spain

**Keywords:** *Balaenoptera*, Baleen whale, Cetacean virology, Common minke whale, Fin whale, Herpesvirus

## Abstract

**Background:**

Herpesvirus can infect a wide range of animal species: mammals, birds, reptiles, fish, amphibians and bivalves. In marine mammals, several alpha- and gammaherpesvirus have been identified in some cetaceans and pinnipeds species. To date, however, this virus has not been detected in any member of the *Balaenoptera* genus.

**Case presentation:**

Herpesvirus was determined by molecular methods in tissue samples from a male fin whale juvenile (*Balaenoptera physalus*) and a female common minke whale calf (*Balaenoptera acutorostrata*) stranded on the Mediterranean coast of the Region of Valencia (Spain). Samples of skin and penile mucosa from the fin whale and samples of skin, muscle and central nervous system tissue from the common minke whale tested positive for herpesvirus based on sequences of the DNA polymerase gene. Sequences from fin whale were identical and belonged to the *Alphaherpesvirinae* subfamily. Only members of the *Gammaherpesvirinae* subfamily were amplified from the common minke whale, and sequences from the muscle and central nervous system were identical. Sequences in GenBank most closely related to these novel sequences were viruses isolated from other cetacean species, consistent with previous observations that herpesviruses show similar phylogenetic branching as their hosts.

**Conclusions:**

To our knowledge, this is the first molecular determination of herpesvirus in the *Balaenoptera* genus. It shows that herpesvirus should be included in virological evaluation of these animals.

## Background

Herpesviruses have been detected in a wide range of hosts, including mammals, birds, reptiles, fish, amphibians and bivalves [1]. The family *Herpesviridae* is divided into three subfamilies: *Alpha*-, *Beta*- and *Gammaherpesvirinae* [2]. All herpesviruses so far detected in marine mammals belong either to the *Alpha*- or *Gammaherpesvirinae* subfamilies [3].

In cetaceans, herpesvirus has been associated with skin lesions [4–7], genital lesions [7–11], nephritis [12], encephalitis [13–15], disseminated infections [16–18], though infection can also occur in the absence of lesions [19]. Moreover, herpesvirus can cause immunosuppression in cetaceans [17], other animal species [20, 21] and humans [22]. Stresses on infected animals, as well as immunosuppression due to non-herpesviral cause, can cause latent herpesvirus to switch to an actively replicating state [23].

Despite analysis of several cases of herpesvirus infection in cetaceans, there is a lack of knowledge about which species are affected and how infection characteristics depend on species, sex or age.

This article reports the molecular detection of three novel herpesviruses in a fin whale (*Balaenoptera physalus*) and a common minke whale (*Balaenoptera acutorostrata*). This is the first molecular determination of herpesvirus from individuals of the *Balaenoptera* genus, indicating that these animals can be infected by herpesvirus.

## Case presentation

On 10 July 2011, a male fin whale stranded at Moncofa on the Mediterranean coast of Castellón in the Region of Valencia, Spain (39°48′31.78″N 0°08′49.40″W). The animal was classified as juvenile based on its length of approximately 900 cm. On 28 April 2014, a female common minke whale stranded at Santa Pola on the Mediterranean coast of Alicante, also in the Region of Valencia (38°12′47.63″N 0°33′40.47″W). The animal was 300 cm long and was classified as a calf, making it the first reported stranding of a common minke whale calf in the Spanish Mediterranean [24].

Both carcasses showed advanced decomposition and were classified as code 4 according to the criteria of Geraci & Lounsbury [25]. This poor state of preservation prevented complete necropsy, histopathology analysis and RNA virus determination. Examination of the corpses failed to show signs of gross lesions, and the cause of death for both animals remains unclear.

The following tissue samples were collected and stored at – 80 °C: skin, blubber and muscle from both animals; penile mucosa and optical nerve from the fin whale; and heart, liver, kidney, ovary and central nervous system (CNS) from the common minke whale.

The Oceanografic is part of the Stranding Network through an agreement between the “Ciudad de las Artes y las Ciencias” and the “Conselleria d’Agricultura, Medi Ambient, Canvi Climàtic i Desenvolupament Rural”. Through this agreement both institutions have transferred to the Oceanografic the rights for veterinary assistance in cases of stranded cetaceans. This agreement includes the rights of participating in the necropsies (together with the University of Valencia) and the use of samples from the cadaveric tissue for research purposes. No samples of blood or tissues were collected from live animals and no animals were euthanized for the purpose of this study. Then, in accordance with the European Parliament and Council normative 2010/63/UE (22nd September 2010) and the Real Decreto 53/2013 (1st February 2013) in post-mortem tissue collection for research purposes, approval from the corresponding ethical committee is not required.

Total DNA was extracted from tissues using the High Pure PCR Template Preparation Kit (Roche Diagnostics, Mannheim, Germany) following the manufacturer’s instructions.

Herpesvirus detection was performed in all collected samples using a panherpesvirus nested polymerase chain reaction (PCR) targeting the DNA polymerase (DNApol) gene [26]. Samples of skin and penile mucosa from the fin whale gave a positive PCR result, as did skin, muscle and CNS samples from the common minke whale. Standard precautions were taken to prevent cross-contamination of samples. Negative controls for DNA extraction and PCR reactions were conducted in the absence of template and using a previously tested negative sample, while positive reactions were conducted using known herpesvirus DNA obtained from walrus [27].

PCR products from all positive samples were purified using the PCR Purification Kit (Qiagen, Germantown, USA) and sequenced. The primers TGVseq, IYGseq, and KG1 [26] were used to obtain longer sequences. The corresponding sequences obtained with these primers covered 200, 410 and 439 base pairs (bp), excluding primers.

Sequence evaluation confirmed herpesvirus infection in both stranded animals. Three novel herpesvirus sequences were found and deposited in GenBank with the following accession numbers: KP995686, amplified from skin and penile mucosa of the fin whale; KP995687, amplified from skin of the common minke whale; and KP995688, amplified from CNS and muscle of the common minke whale.

These sequences were subjected to phylogenetic analysis using MEGA 6.0 software [28], based on the 200-bp region amplified for the three herpesviruses. The accuracy of the alignment, which reflects the reliability of the resulting phylogenetic tree, was assessed by calculating the average amino acid identity and then calculating the average p-distance (1 - amino acid identity). Since the average p-distance (0.5329) was below the recommended threshold of 0.8 [29, 30], the alignment was considered acceptable. In addition, the average pairwise Jukes-Cantor distance (0.8707) was below the threshold of 1.0 [31], suggesting that the data were adequate for generating neighbor-joining trees.

**Fig. 1:**
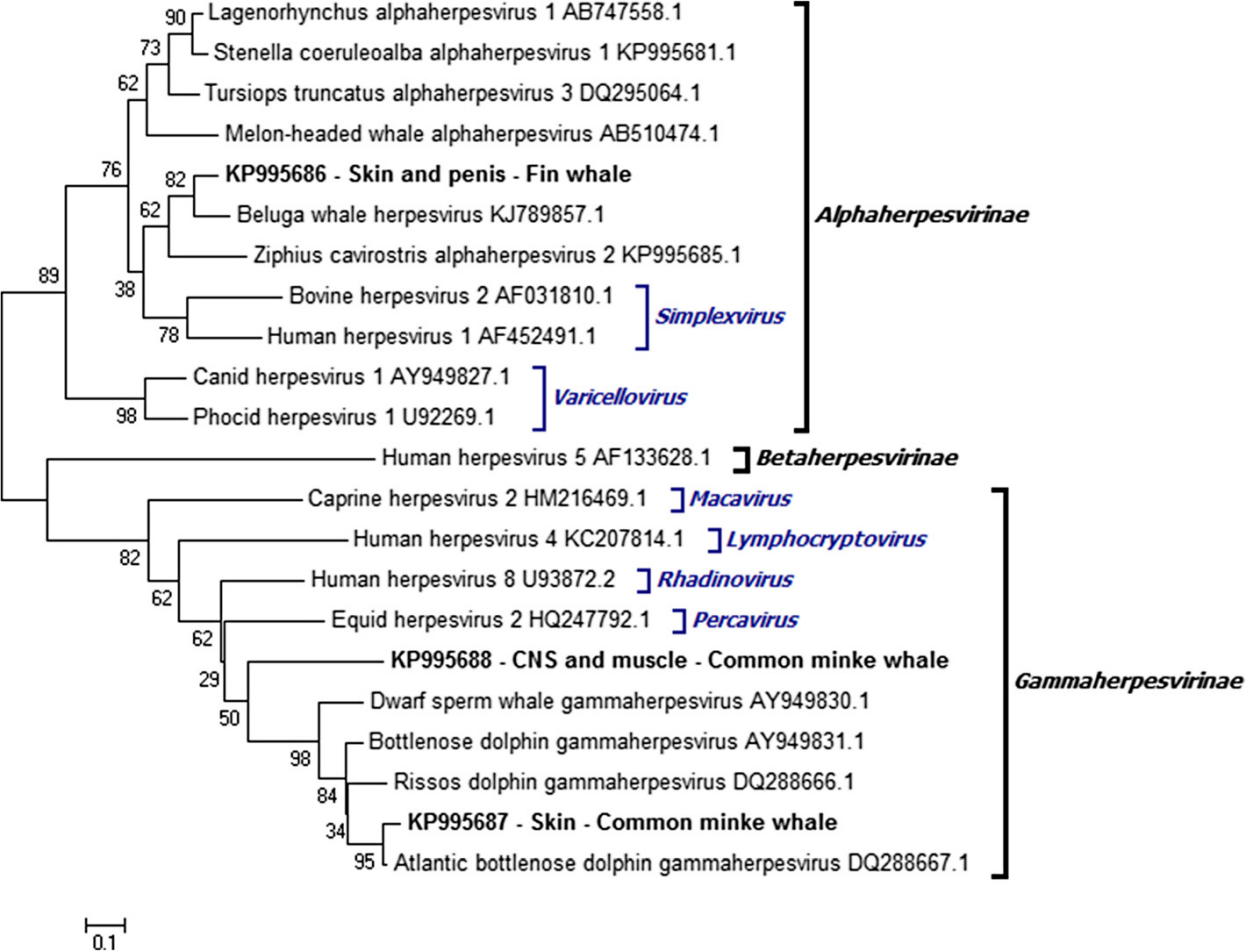
Phylogram representing relationships among viruses from *Balaenoptera physalus*, *Balaenoptera acutorostrata* and other hosts. A phylogenetic tree was inferred using the amino acid sequences encoded by the herpesvirus DNA polymerase gene. The reliability of the neighbor-joining (NJ) tree topology was tested by bootstrapping 2000 replicates generated with a random seed, and results are indicated at the tree nodes. The bar at the bottom indicates relative phylogenetic distance. Each sequence is named according to the virus name and GenBank accession number. Boldfaced sequences were amplified from *B. physalus* skin and penile mucosa (KP995686), or from *B. acutorostrata* skin (KP995687) and central nervous system or muscle (KP995688). These sequences are labelled as “*GenBank accession number* - *tissue samples* - *host*”. Herpesvirus genera are indicated according to Davison [2]. The amino acid p-distance of the phylogenetic tree was within a preestablished threshold, indicating its reliability, while the average pairwise Jukes-Cantor distance for the sequence alignment was also within a preestablished threshold, indicating that it could be used to construct NJ trees [29–31]. NJ trees were strictly bifurcated and bootstrap values lower than 50 were considered to be polytomies. Since this cut-off value for defining polytomies is arbitrary, we decided to show all bootstrap values rather than collapse the branches

A phylogenetic tree based on amino acid sequences was constructed within MEGA 6.0 using the neighbor-joining (NJ) statistical method with the Jones-Taylor-Thornton (JTT) model [32]. A bootstrap test of 2000 replications was performed to estimate tree reliability. In order to root the phylogram, a betaherpesvirus sequence was used as outgroup (Fig. 1).

Phylogenetic analysis showed that the sequence obtained from skin and penile mucosa of the fin whale (KP995686) belonged to the *Alphaherpesvirinae* subfamily (Fig. 1). The sequences most closely related to this novel sequence were from beluga whale herpesvirus (KJ789857.1; amino acid p-distance, 0.1377) and ziphius cavirostris alphaherpesvirus (KP995685.1; 0.2656).

Sequences obtained from the CNS and muscle of the common minke whale were identical (KP995688), but different from the sequence detected in skin (KP995687; amino acid p-distance, 0.4237). Both sequences belonged to the *Gammaherpesvirinae* subfamily (Fig. 1). The sequence from the CNS and muscle differed substantially from those in GenBank, showing the closest relationship to sequences from bottlenose dolphin gammaherpesvirus (AY949831.1; 0.3609), Atlantic bottlenose dolphin gammaherpesvirus (DQ288667.1; 0.3636), dwarf sperm whale gammaherpesvirus (AY949830.1; 0.3684), equid herpesvirus 2 (HQ247792.1; 0.3750), Risso’s dolphin gammaherpesvirus (DQ288666.1; 0.3788) and human herpesvirus 8 (U93872.2; 0.3971). GenBank sequences showing the closest relationship to the sequence from the skin of the common minke whale were from Atlantic bottlenose dolphin gammaherpesvirus (DQ288667.1; 0.0667), bottlenose dolphin gammaherpesvirus (AY949831.1; 0.1833) and Risso’s dolphin gammaherpesvirus (DQ288666.1; 0.2167).

The novel herpesvirus DNApol sequences described here from fin whale and common minke whale were most closely related to DNApol sequences from other cetacean herpesviruses. This is consistent with previous observations that herpesvirus phylogenetic branching resembles that of its hosts [27, 33–35].

Histopathological analysis of tissues with herpesvirus infection could not be performed because of the advanced stage of decomposition. The tissues in which herpesvirus was found (skin, genital mucosa and CNS) are also mentioned in most previous reports of herpesvirus in cetaceans [4–11, 13–15]. Herpesvirus infection in these tissues may limit reproduction and cause fatal encephalitis. Nevertheless, herpesvirus infection can occur in the absence of lesions [19], and virus can enter a period of latency [36, 37].

Although our inability to perform histopathology means we do not know whether herpesvirus infection in these two stranded animals was associated with microscopic lesions or not, our results nevertheless clearly demonstrate that herpesvirus can infect members of the *Balaenoptera* genus. Therefore herpesvirus should be taken into account during pathological evaluation of these species.

## Conclusion

Novel alpha- and gammaherpesvirus have been amplified from a fin whale and a common minke whale, and these viruses show the closest phylogenetic relationships to viruses isolated from other cetacean species. To the best of our knowledge, this is the first report of herpesviruses from these baleen whale species, as well as the first molecular determination of herpesvirus in the *Balaenoptera* genus. Therefore, herpesvirus should be included in the virological evaluation in these species.

## Abbreviations

Bp: base pairs
CNS: central nervous system
DNApol: DNA polymerase
JTT: Jones-Taylor-Thornton
NJ: Neighbor Joining
PCR: polymerase chain reaction
